# Successful resuscitation of acute type A aortic dissection with pulmonary embolism using long-term venoarterial extracorporeal membrane oxygenation: a case report

**DOI:** 10.3389/fmed.2025.1530566

**Published:** 2025-01-29

**Authors:** Guiwei Zhu, Wenhui Yue, Yanmin Li, Haiqing Wang, Jianqiang Li, Xin Zhou, Qinghai Zhang, Jihong Zhang

**Affiliations:** ^1^Department of Critical Care Medicine, Weifang People’s Hospital, Weifang, Shandong; ^2^Department of Hand and Foot Orthopedic Surgery, Weifang People’s Hospital, Weifang, Shandong, China; ^3^Office of Medical Affairs, Weifang People’s Hospital, Weifang, Shandong, China

**Keywords:** acute type A aortic dissection, pulmonary embolism, veno-arterial extracorporeal membrane oxygenation, cardiopulmonary arrest, hemodynamic instability, case report

## Abstract

Acute aortic dissection (AAD) and pulmonary embolism (PE) are two critical and potentially fatal causes of chest pain. The simultaneous occurrence of these conditions is exceptionally rare; however, when they co-occur, the conflicting therapeutic strategies required significantly elevate mortality rates. While venoarterial extracorporeal membrane oxygenation (VA-ECMO) has been extensively reported as a life-saving intervention for high-risk pulmonary embolism, its application in the management of AAD remains highly debated. Recently, our institution successfully employed VA-ECMO to treat a patient with acute type A aortic dissection (ATAAD) complicated by PE. This case highlights that VA-ECMO may serve as a crucial life-saving measure for patients with AAD who present with hemodynamic instability or cardiac arrest.

## Introduction

Acute aortic dissection (AAD) is characterized by a disruption of the tunica intima, allowing blood to enter the tunica media, which can propagate along the length of the aorta and extend into its branch vessels. For patients with acute type A aortic dissection (ATAAD) managed conservatively, the 24-h mortality rate is approximately 60% (1). Pulmonary embolism (PE) is the third leading cause of cardiovascular death, with 30-day and 6-month all-cause mortality rates of 9.1 and 19.6%, respectively, in the Medicare population (2). The simultaneous occurrence of AAD and PE is exceedingly rare; however, their concurrent presentation significantly amplifies mortality due to conflicting therapeutic strategies. While venoarterial extracorporeal membrane oxygenation (VA-ECMO) is well-documented as a life-saving intervention for high-risk PE (3), its application in AAD remains highly debated, as AAD is considered a relative contraindication for VA-ECMO (4). To date, only one case has been reported in which VA-ECMO was utilized in the management of a type B aortic dissection complicated by PE (5), and no cases have been documented for ATAAD with concurrent PE.

Here, we present the case of a patient with a malignant tumor in the upper lobe of the left lung (pT1N0M0, stage IA). On the first postoperative day following lobectomy, the patient experienced cardiopulmonary arrest during ambulation. Despite cardiopulmonary resuscitation, stable autonomous cardiac rhythm could not be achieved, necessitating the initiation of VA-ECMO therapy. Comprehensive diagnostic evaluation subsequently revealed ATAAD complicated by PE. Following 27 h of intensive management, VA-ECMO support was successfully weaned, and the patient underwent surgical intervention for ATAAD. The patient achieved full recovery, with no residual symptoms observed. This case represents the first successful use of VA-ECMO in the management of a patient with ATAAD complicated by PE.

## Case description

A 60-year-old female (height: 165 cm, weight: 64.5 kg) underwent a routine chest CT scan 2 years and 5 months prior, revealing ground-glass nodules in the right upper lobe and bilateral lower lobes. No specific treatment was administered at the time. However, a follow-up chest CT scan conducted 15 days before admission identified a mixed ground-glass nodule in the apicoposterior segment of the left upper lobe, prompting her presentation to our hospital for further evaluation and management. The patient reported no symptoms such as persistent or irritative cough, sputum production, hemoptysis, fever, night sweats, or fatigue. She denied chest pain, tightness, dyspnea, hoarseness, or dysphagia. Her past medical history was unremarkable, with no history of occupational or toxic substance exposure, and no history of smoking, alcohol use, or drug abuse.

On physical examination, vital signs were stable, including a temperature of 36°C, pulse of 76 beats per minute, respiratory rate of 19 breaths per minute, and blood pressure of 120/99 mmHg. There was no palpable lymphadenopathy in the supraclavicular regions, and the thorax appeared symmetrical with normal bilateral respiratory movement. Percussion revealed resonance bilaterally, and auscultation noted clear breath sounds without adventitious sounds. Cardiovascular examination showed a regular heart rate of 76 beats per minute with no pathological murmurs in any valve region. The remainder of the systemic examination was unremarkable.

Upon admission, comprehensive diagnostic tests were performed. Echocardiography revealed mild tricuspid regurgitation but no other abnormalities. Venous ultrasonography of the bilateral common iliac, internal and external iliac, femoral, popliteal, anterior and posterior tibial, and intermuscular veins showed no evidence of thrombus. Contrast-enhanced chest CT demonstrated a ground-glass nodule in the left upper lobe, raising suspicion for a neoplastic lesion, along with bilateral ground-glass micronodules, solid micronodules, and a calcified nodule in the right upper lobe.

**FIGURE 1:**
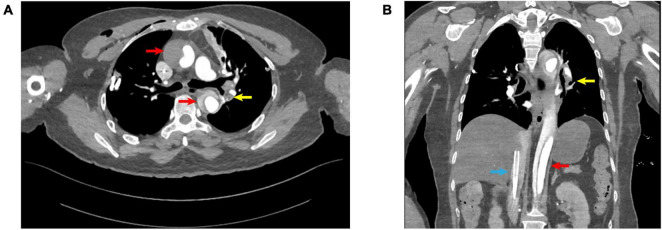
**(A)** Transverse view; **(B)** coronal view; red arrows: aortic dissection; yellow arrows: pulmonary embolism; blue arrows: V-A ECMO venous drainage cannula.

On the second day of hospitalization, the patient underwent a “right lung nodule microwave ablation,” with an uneventful postoperative course. On the sixth day of hospitalization, she underwent a “thoracoscopic resection of LS(1+2)(a+b)+3c segments with lymphadenectomy.” Postoperative histopathological examination of the lung tissue revealed a moderately differentiated invasive adenocarcinoma, with no evidence of lymph node metastasis. On the first postoperative day (seventh day of hospitalization) at 14:30, during her initial ambulation attempt, the patient experienced transient syncope characterized by pallor, diaphoresis, and dyspnea. She remained responsive. Oxygen therapy at 6 L/min via a face mask was immediately initiated, and peripheral blood glucose measured 7.3 mmol/L. Cardiac monitoring showed a heart rate of 123 beats per minute, respiratory rate of 36 breaths per minute, oxygen saturation of 85%, and blood pressure of 82/43 mmHg. Auscultation revealed coarse bilateral breath sounds. Intravenous access was established, and 500 mL of lactated Ringer’s solution was administered. The patient’s condition rapidly deteriorated, and at 14:40, cardiac monitoring revealed pulseless electrical activity. Immediate chest compressions were initiated, followed by intravenous epinephrine administration, bedside endotracheal intubation, and manual ventilation. Spontaneous cardiac activity resumed at 14:45; however, hemodynamic stability could not be achieved despite high-dose norepinephrine (1.68 μg/kg/min). Bedside ultrasonography revealed right ventricular dilation and global hypokinesia. Considering that the patient was on the first postoperative day after a lung lobectomy and had contraindications to thrombolytic therapy, we opted to initiate VA-ECMO to stabilize her circulation. Ultrasound-guided cannulation of the left femoral vein and right femoral artery was performed, with successful puncture of both vessels on the first attempt. VA-ECMO was successfully commenced at 15:41. Under VA-ECMO support, computed tomography angiography (CTA) identified ATAAD with concurrent PE (Figures 1A, B). Multidisciplinary discussion determined that, due to the small size of the PE, thrombolysis was unnecessary. Anticoagulation therapy was initiated with a target activated clotting time of 180–220 s. VA-ECMO flow was progressively reduced while monitoring hemoglobin levels and coagulation parameters. As the hospital lacked surgical capacity for ATAAD, and the risks of transporting the unstable patient were significant, arrangements were made with the family to transfer the patient to a higher-level facility once her vital signs stabilized.

After 27 h on VA-ECMO, the patient regained consciousness, achieved hemodynamic stability, and no longer required vasopressor support. VA-ECMO was successfully weaned and explanted, and she was transferred to a higher-level hospital on the same day. At the receiving facility, the patient underwent a “aortic valve repair, partial ascending aortic resection with graft replacement, total aortic arch replacement, and stented elephant trunk procedure (Sun’s procedure).” Intraoperative findings revealed the dissection tear on the lesser curvature of the aortic arch. Cardiopulmonary bypass was discontinued without complications.

**FIGURE 2:**
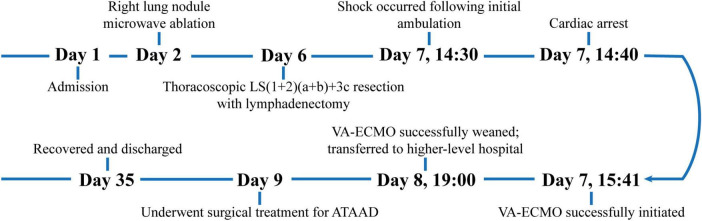
The timeline of the patient’s disease progression.

The patient’s postoperative course was favorable, and she was discharged in stable condition. At her 9-month follow-up visit, she exhibited no neurological deficits, all organ functions were normal, and pulmonary CTA showed no evidence of thrombus formation (Figure 2).

## Discussion

Acute aortic dissection is characterized by a disruption of the tunica intima, allowing blood to enter the tunica media, which can propagate along the entire length of the aorta and its branch vessels. The incidence of AAD is estimated to be 5–30 cases per million individuals annually. ATAAD involves the ascending aorta, is highly fatal if left untreated, with a 24-h mortality rate of approximately 60% in conservatively managed cases. Mortality increases substantially when complications such as cardiac tamponade (with or without cardiogenic shock), acute myocardial ischemia or infarction, stroke, or malperfusion of vital organs occur (1). PE is the third leading cause of cardiovascular death, with 30-day and 6-month all-cause mortality rates of 9.1 and 19.6%, respectively, in the Medicare population (2). The simultaneous occurrence of AAD and PE is exceedingly rare; however, when these conditions co-occur, the conflicting management strategies significantly increase mortality. VA-ECMO has been extensively reported as a rescue therapy for high-risk PE (3), but its use in AAD remains controversial due to the risk of exacerbating the dissection, AAD is generally considered a relative contraindication for VA-ECMO (4). To date, only one case has been reported describing VA-ECMO use in type B aortic dissection complicated by PE (5), with no documented cases involving ATAAD with concurrent PE. At our institution, VA-ECMO was successfully utilized to manage a patient with ATAAD complicated by PE. This represents the first documented case of successful VA-ECMO intervention in a patient with ATAAD and concurrent PE.

The precise temporal relationship and potential interactions between ATAAD and PE in this case remain challenging to ascertain. Aoki et al. previously reported a case of ATAAD caused by adjustments to the position of the VA-ECMO return cannula (6). However, in our patient, the dissection tear was located on the lesser curvature of the aortic arch, making VA-ECMO cannulation an unlikely cause of the ATAAD.

One of the primary concerns regarding VA-ECMO use in patients with ATAAD is the risk of arterial cannulation inadvertently entering the false lumen. Current protocols for arterial cannulation during cardiopulmonary bypass in ATAAD include options such as the ascending aorta, innominate artery, axillary artery, central artery, and femoral artery. While the axillary artery is preferred for its ability to maintain adequate cerebral perfusion, femoral artery cannulation remains the first choice for hemodynamically unstable patients due to its ease and rapid accessibility (7). Data from the German Registry for Acute Aortic Dissection Type A (GERAADA) indicate that only 50.1% of ATAAD cases involve the descending aorta, with 37.9% involving the abdominal aorta and 24.8% involving the pelvic arteries (8). Therefore, in at least 75% of cases, femoral artery cannulation for VA-ECMO is unlikely to result in false lumen perfusion. Furthermore, ultrasound-guided puncture can minimize cannulation errors, making it a safe and feasible option for hemodynamically unstable patients. Concerns about potential cannulation into the false lumen should not preclude the use of this life-saving intervention.

For high-risk PE, current guidelines recommend anticoagulation combined with thrombolytic or thrombectomy therapy (2). However, in this case, the concurrent ATAAD posed a significant bleeding risk. Given the small burden of PE on pulmonary CTA, thrombolysis or thrombectomy was deemed unnecessary. Anticoagulation therapy in patients with combined AAD and PE lacks standardized protocols. We opted for unfractionated heparin, given its ability to be rapidly neutralized with protamine, targeting an activated clotting time ACT of 180–220 s. Platelet counts were maintained within normal limits, and hemoglobin levels were closely monitored. To prevent retrograde VA-ECMO flow from exacerbating hematoma expansion or causing rupture of the dissection, blood pressure was meticulously controlled within a target range of 100–120 mmHg during VA-ECMO support. VA-ECMO flow was gradually reduced as the patient’s hemodynamics stabilized. No expansion of the dissection hematoma was observed, and VA-ECMO was successfully weaned without complications.

ATAAD necessitates urgent surgical intervention, with literature indicating an approximately 1–2% increase in mortality per hour without surgery, reaching up to 60% within 24 h (1). However, due to the lack of surgical capabilities at our facility, emergency surgery could not be performed, and VA-ECMO was utilized for 27 h. This represents the longest documented duration of VA-ECMO support for a patient with AAD. Malperfusion of the heart and visceral organs in aortic dissection is associated with poor outcomes, as irreversible organ damage significantly increases mortality. Recent studies suggest that restoring end-organ perfusion through endovascular techniques prior to aortic repair may improve outcomes (7). Hiroyuki Ohbe et al. demonstrated that extracorporeal cardiopulmonary resuscitation can improve outcomes in some patients with AAD complicated by refractory cardiac arrest (9). Although current evidence remains insufficient to overturn the contraindication of VA-ECMO in AAD, further clinical studies are needed to evaluate its viability as a rescue therapy in this population. In cases of refractory cardiac arrest of unknown origin, VA-ECMO should be promptly considered when patients meet specific criteria (10).

## Conclusion

For patients with AAD presenting with hemodynamic instability or cardiac arrest, VA-ECMO may serve as a critical life-saving intervention.

## Data Availability

The original contributions presented in this study are included in this article/Supplementary material, further inquiries can be directed to the corresponding author.
